# Construction of a high-density linkage map and fine mapping of QTL for growth in Asian seabass

**DOI:** 10.1038/srep16358

**Published:** 2015-11-10

**Authors:** Le Wang, Zi Yi Wan, Bin Bai, Shu Qing Huang, Elaine Chua, May Lee, Hong Yan Pang, Yan Fei Wen, Peng Liu, Feng Liu, Fei Sun, Grace Lin, Bao Qing Ye, Gen Hua Yue

**Affiliations:** 1 Temasek Life Sciences Laboratory, National University of Singapore, 1 Research Link, Singapore 117604; 2 Department of Biological Sciences, National University of Singapore, 14 Science Drive, Singapore 117543; 3 School of Biological Sciences, Nanyang Technological University, 6 Nanyang Drive, Singapore 637551.

## Abstract

A high-density genetic map is essential for comparative genomic studies and fine mapping of QTL, and can also facilitate genome sequence assembly. Here, a high density genetic map of Asian seabass was constructed with 3321 SNPs generated by sequencing 144 individuals in a F_2_ family. The length of the map was 1577.67 cM with an average marker interval of 0.52 cM. A high level of genomic synteny among Asian seabass, European seabass, Nile tilapia and stickleback was detected. Using this map, one genome-wide significant and five suggestive QTL for growth traits were detected in six linkage groups (i.e. LG4, LG5, LG11, LG13, LG14 and LG15). These QTL explained 10.5–16.0% of phenotypic variance. A candidate gene, *ACOX1* within the significant QTL on LG5 was identified. The gene was differentially expressed between fast- and slow-growing Asian seabass. The high-density SNP-based map provides an important tool for fine mapping QTL in molecular breeding and comparative genome analysis.

A linkage maps is an essential tool for mapping QTL for important traits for molecular breeding of economically important animal and plant species. Construction of a high-density genetic linkage map provides useful genomic resources for fine-scale mapping of quantitative trait loci (QTL) and positional cloning of genes controlling the important traits^1^,^2^,^3^,^4^. It also allows for characterization of recombination hotspots along individual chromosomes^5^. In addition, high-density genetic maps constructed using sequence-based markers are particularly preferred for comparative genomic analysis to study chromosomal organization and evolution^5^. Last but not least, a dense sequence-based genetic linkage map facilitates *de novo* genome assembly at chromosomal level^6^,^7^.

Obtaining a large number of genetic markers and conducting cost-effective genotyping in populations are essential prerequisites for construction of a high-density linkage map. For most non-model species, however, it is still impossible to carry out affordable large scale marker discovery and array-based genotyping in the same way as in model species^8^. Although possessing the advantages of high level of polymorphisms and low cost of genotyping for microsatellites, the wet lab work of genotyping microsatellites to construct a high-density genetic mapping is laborious and tedious. Recent advances in the next-generation sequencing technologies (NGS) have resulted in rapid and cost-effective massive marker discovery and genotyping in both model and non-model species, mainly in the form of single nucleotide polymorphisms (SNP)^8^. Several genotyping by sequencing (GBS) approaches have been developed^9^,^10^,^11^, which have significantly reduced the cost of genotyping and labour and made it possible for parallel genotyping tens of thousands of SNP markers in non-model species. For these GBS approaches, restriction site-associated DNA (RAD) libraries need to be constructed and sequenced using NGS platforms to discover and genotype a large number of genetic markers shared by each individual^9^,^10^,^11^.

To date, genetic maps have been constructed in over 45 fish species^1^. However, most of the genetic maps were constructed using amplified-fragment length polymorphism markers (AFLP) and microsatellites. The density of these linkage maps in aquaculture species is still low. In some species, such as Atlantic salmon (*Salmo salar*)^12^, Japanese flounder (*Paralichthys olivaceus*)^13^, European seabass (*Dicentrarchus labrax*)^14^, Asian seabass^15^ and channel catfish (*Ictalurus punctatus*)^16^, medium-density linkage maps were constructed with microsatellites and some SNPs. With the development of NGS technologies and RAD-based genotyping approaches, high-density SNP-based linkage maps have been constructed for several economically important fish species, e.g. Atlantic salmon^5^, channel catfish^17^, and Japanese flounder^6^. Compared to traditional low-density genetic maps, these high-density genetic maps have shown significant advantages in fine mapping of QTL, comparative genomics and facilitating *de novo* genome sequence assembly.

Asian seabass, *Lates calcarifer* is the most important marine food fish species in Southeast Asia and Australia. It has been cultured for nearly 30 years in these regions^15^. For the past decades, various genetic resources have been developed in our group to facilitate the breeding program of this species, such as hundreds to thousands of microsatellite and SNP markers^15^,^18^,^19^,^20^,^21^, linkage and physical maps^15^,^22^,^23^ and cDNA and BAC libraries^24^,^25^,^26^. Using these resources, we have conducted QTL mapping and association studies and identified several candidate loci and genes responsible for growth and production traits^15^,^27^,^28^,^29^ and disease resistance^30^,^31^. However, the low density of markers in our previous linkage maps, particularly in the QTL regions, limited the potential to fine map QTL for important traits and to search for the candidate genes.

The purposes of this study were to construct a high-density SNP-based linkage map and to fine map QTL for growth traits to facilitate genetic improvement in Asian seabass and to identify potential genes determining growth traits. We constructed a linkage map with 3321 SNPs using genotyping by sequencing on an Illumina NextSeq500 platform. We identified a major QTL for growth traits on linkage group (LG) 5. A potential candidate gene for growth, *ACOX1*, was identified. The high-density SNP-based linkage map provides an essential tool in dissecting QTL and identifying candidate genes underlying complex traits. The map is also useful in facilitating future assembly of the Asian seabass genome and comparative genomics studies.

## Results

### SNP genotyping by sequencing

A total of 888.47 million clean reads were produced by the NextSeq500 platform after sequential quality filtering and sequence trimming, within which 31.06 and 23.87 million reads were produced for parents, respectively. The average clean reads for each progeny was 5.87 million. Using the data sets of the parental samples, a catalogue containing 101035 loci was constructed. This catalogue was used as reference for SNP discovery and genotyping in the mapping family using the program *genotypes*. 18262 SNP markers were identified across all the samples, within which, 3928 SNPs were genotyped in more than 80% of progenies. All these genotyped SNPs were used for linkage mapping.

### Construction of linkage maps

**Figure 1:**
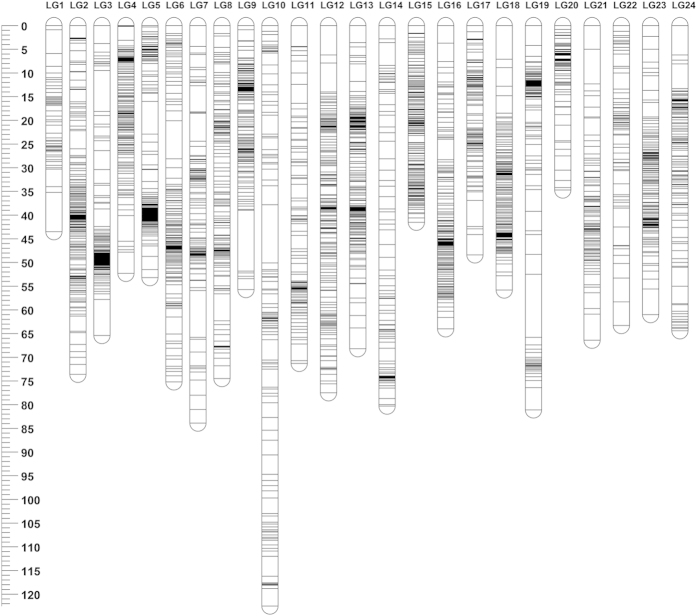
Genetic lengths and marker distribution of 24 linkage groups in the sex-averagedlinkage map of Asian seabass.

After removal of the markers with distorted segregation, 3349 markers were retained. Among these markers, 1912 and 2095 SNPs were heterozygous in paternal and maternal samples, respectively, while 658 markers were heterozygous in both parents. For the sex-averaged linkage map, a total of 3321 SNPs were successfully mapped into 24 LGs (Fig. 1 and Table 1). The total genetic length of the sex-averaged linkage map was 1577.67 cM. The genetic length of individual LGs ranged from 34.71 cM for LG20 to 122.49 cM for LG10, with an average of 65.74 cM. The marker interval was from 0.23 cM for LG15 to 1.08 cM for LG10, with an average of 0.52 cM. The largest gap was located in LG19 with a genetic length of 13.30 cM. All the markers with flanking sequences are listed in Supplementary Table S1. For the sex-specific linkage maps, 2077 and 1898 SNPs were mapped into the female- and male-specific maps, respectively, with 24 LGs in each map (Supplementary Figure S1, Figure S2 and Table S2). The total genetic distance of the female-specific linkage map was 1496.43 cM, with linkage groups ranging from 33.79 cM for LG4 to 94.44 cM for LG12, and with an average of 62.36 cM. The marker interval of this map ranged from 0.44 cM for LG15 to 1.50 cM for LG1, with an average of 0.81 cM. The corresponding male-specific map had a total genetic length of 1324.39 cM. The length of individual LGs ranged from 11.26 cM for LG22 to 82.60 cM for LG12, with an average of 55.18 cM. The marker interval for individual LGs ranged from 0.38 cM for LG22 to 3.41 cM for LG14, with an average of 0.90 cM. The female to male length ratio for each group ranged from 0.64 for LG4 to 6.45 for LG22. Detailed information of all the linkage maps is listed in Supplementary Table S3.

### Recombination rates between sexes

Comparisons between the sex-specific maps revealed significant differences in recombination rates and the distribution of recombination events between the two sexes. First of all, there was a difference in the length between female- and male-specific genetic map, with an average female to male ratio of 1.13 (Supplementary Table S2). LGs LG14, LG19 and LG22 showed much higher recombination rates in females than in males, whereas LG2, LG4 and LG15 were identified to have more frequent recombination in males (Supplementary Table S2). The distributions of recombination events tended to concentrate towards the ends of each LG (Supplementary Figure S3). Only LG4 and LG12 of the female-specific map, and LG9 and LG22 of both sex-specific maps, showed relatively frequent recombination in the middle of each linkage group (Supplementary Figure S3). We also observed that recombination events tended to occur near the centromeres rather than the telomeres. For several LGs, such as LG7, LG8, LG10, LG15, LG16, LG17 and LG23, little difference was observed in the distribution patterns of recombination events (Supplementary Figure S3). Significant differences in the distribution patterns of recombination between female and male were found in LG2, LG4, LG6, LG9, LG12, LG13 and LG19 (Supplementary Figure S3).

### Annotation of mapped markers and identification of genomic synteny

Sequence alignment to reference genomes and Blast2GO analysis annotated a total of 1860 of the SNP markers (56.0%), which were located in the exonic and intronic regions of the genomes and associated with a putative gene (Supplementary Table S4). Over 50% of markers were annotated for almost all the 24 LGs except for LG10, LG16 and LG17 (Supplementary Figure S4).

**Figure 2:**
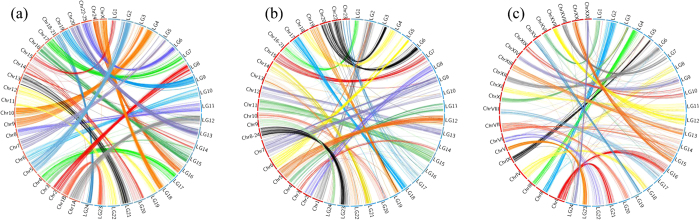
Genomic synteny as shown by Circos diagram for each pair of alignments between Asian seabass and (**a**) European seabass, (**b**) Nile tilapia and (**c**) stickleback.

We investigated the genomic synteny between Asian seabass and other model and non-model fish species by anchoring the marker sequences of Asian seabass to the chromosomes of those species. Within perciforms, 1540 (46.4%) and 1037 (31.2%) markers of Asian seabass were uniquely mapped to the reference genome of European seabass and Nile tilapia, respectively. A high level of genomic synteny was observed between Asian seabass and European seabass, with 1515 (98.4%) aligned segments located into syntenic boxes at the chromosomal level (Supplementary Figure S5). A 1:1 relationship was observed between Asian seabass LGs and European seabass chromosomes except for chromosome 14 in European seabass, which showed synteny to LG14 and LG20 in Asian seabass (Supplementary Figure S5 and Fig. 2a). The overall level of genomic synteny between Asian seabass and Nile tilapia was also high, with 986 (95.1%) aligned segments located into syntenic boxes (Supplementary Figure S5). Four LGs (LG9, LG10, LG16 and LG17) in Asian seabass were mapped to two chromosomes (chromosome 7 and 23) of Nile tilapia, while the others showed a 1:1 syntenic relationship for each pair of LGs (Supplementary Figure S5 and Fig. 2b). Comparisons to model fish species revealed a high but slightly reduced degree of conserved genomic synteny between Asian seabass and stickleback, with 912 (27.5%) conserved markers identified between the two species and 828 (90.8%) aligned segments located into the syntenic boxes (Supplementary Figure S5 and Fig. 2c). Six LGs (LG16, LG21, LG8, LG18, LG14 and LG20) in Asian seabass merged into three chromosomes (chromosome I, IV and VII) in stickleback. However, we did not identify a conserved genomic synteny between Asian seabass and zebrafish, with only 5.6% shared markers between the two species. The 1:1 syntenic relationship for each marker, across all the LGs and chromosomes, between Asian seabass and European seabass, Nile tilapia and stickleback is visualized in Fig. 2.

### Fine mapping of QTL and preliminary characterization of a candidate gene *ACOX1*

**Figure 3:**
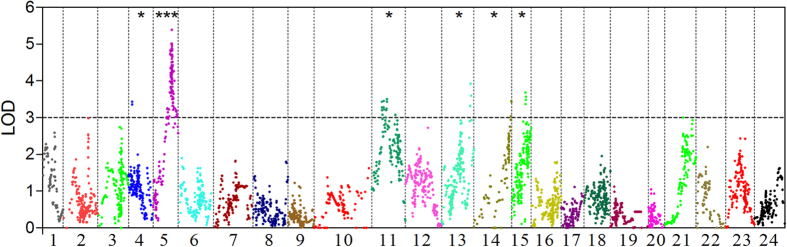
Whole genome scan of QTL for growth-related traits in Asian seabass, where chromosome-wide LOD (3.0) threshold is denoted with dashed line. Significance for each QTL region is denoted with *(chromosome-wide *P* < 0.05) and ***(genome-wide *P* < 0.01).

**Figure 4:**
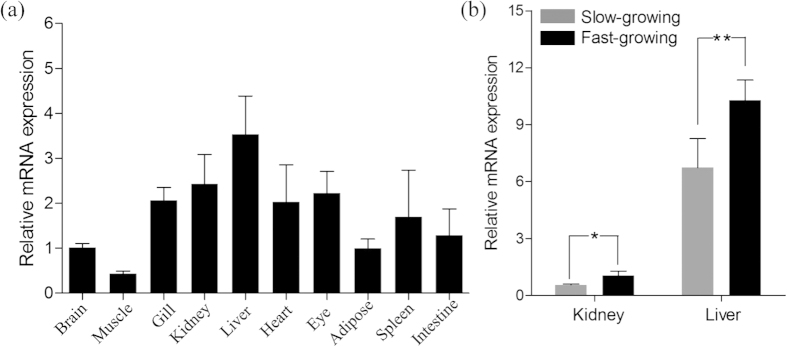
The expression pattern of *ACOX1* gene in (**a**) 10 tissues and (**b**) between two fish groups of different body weight of Asian seabass. *and **denote significance level of *P* < 0.05 and 0.01, respectively.

Pearson’s test revealed that strong correlation coefficients (R value from 0.952 to 0.981, *P* < 0.001) between each pair of growth traits. Genome scans of QTL for these traits showed similar results in terms of LOD profile and its significance threshold. Therefore, only the results of body weight are reported here. Five suggestive QTL in LG4, LG11, LG13, LG14 and LG15 were identified, explaining 10.5%, 10.7%, 11.9%, 10.5% and 11.3% of the total phenotypic variations, respectively (Fig. 3 and Table 2). One significant QTL, explaining 16.0% of the total phenotypic variations, was detected in LG5 (Fig. 3 and Table 2). The significant confidence intervals of the five suggestive QTL spanned a relatively narrow region of a maximum of 1.7 cM. The nearest SNP to the peak of each suggestive QTL region is listed in Table 2. The confidence interval of the significant QTL in LG5 covered a region of 5.6 cM and 0.7 cM at the significance level of 0.05 and 0.01, respectively. Within the region of 0.7 cM, 18 SNP markers were mapped and six genes were identified based on the genomic synteny between Asian seabass and European seabass and between Asian seabass and Nile tilapia using these markers. Among these genes, Peroxisomal acyl-coenzyme a oxidase 1 (*ACOX1*) was located closest to the peak of this QTL region (LOD, 5.39). Expressions of *ACOX1* were found in all 10 examined tissues (Fig. 4a). The highest expression was observed in the liver, followed by the kidney. The expression level of *ACOX1* was significantly higher in fast-growing fish than in slow-growing fish in both the liver (1.5 fold, *P* < 0.01, two-tailed t-test) and kidney (1.8 fold, *P* < 0.05, two-tailed t-test) (Fig. 4b).

## Discussion

High-density linkage maps, especially those constructed using sequence-based SNP markers, are useful in genetic studies including comparative genomics, fine mapping of interesting genes, positional cloning of candidate genes and facilitating assembly of genome sequences^1^,^2^,^3^,^4^. To date, SNP microarray is the common approach for genetic map construction in model species^32^, while RAD sequencing approaches are becoming popular for linkage mapping in non-model species^5^,^6^. Here, we used the ddRAD-seq approach to discover and genotype SNPs in Asian seabass and constructed a linkage map using 3928 RAD-based SNP markers. This approach has been used in linkage map constructions for some model and non-model species and shown robust confidence in SNPs genotyping^9^,^33^. Although we used *de novo* assembly of stacks in the process of SNP discovery due to lack of genome information for Asian seabass, the accuracy of SNP discovery and genotyping was similar to the reference-based method^9^,^34^. Therefore, our SNP genotyping data is robust and can be used in constructing a high-density linkage map.

The second-generation genetic map of Asian seabass was constructed using 790 microsatellites and SNPs^15^. However, the markers were unevenly distributed across several LGs. Some LGs were constructed using only a dozen markers and there were also some large gaps of up to 45 cM^15^. The map was not sufficient for fine mapping QTL for marker-assisted selection (MAS). In this study, a sex-averaged genetic map with 3321 SNPs was constructed. The LGs of this map are in full agreement with the linkage maps previously published by us^15^,^23^. This map has a total length of 1577.67 cM with an average marker interval of 0.52 cM, much denser than our previous maps^15^,^23^. The current high-density linkage map has substantially improved previous maps of Asian seabass^23^,^27^, thus supplying an important tool for fine mapping QTL for important traits. In addition, the SNP-based high-density linkage map can be used to anchor sequence scaffolds in the assembly of whole genome sequence of Asian seabass and comparative genomic studies. However, we noticed that in some LGs of the current map, some marker gaps (i.e. 13.30 cM in LG19) were still big. Therefore, more markers should be used in the future.

The total length of the female map was slightly longer than that of the male map (1496.63 cM vs 1324.39 cM). This pattern of sex difference in recombination has been identified in Asian seabass^15^ and in other fish species^5^. In addition, significant differences in length were also identified for individual LGs, where female LGs are much longer than those of males, suggesting less frequent recombination events during meiosis in males than in females. We also observed significant differences in the distribution of recombination events between sexes in several linkage groups. Recombination events were found to be more frequent towards telomeres while recombination was suppressed around the centromeres for both sexes. This is a common phenomenon in fish species like Japanese eel (*Anguilla japonica*)^33^, channel catfish^17^ and Atlantic salmon^5^. Such a pattern of uneven distribution of recombination along LGs is likely caused by specific sequences and genomic structure of the chromosomes.

A high-density genetic map constructed with sequence-based markers makes it possible for comparative genomic studies in non-model species without genome information^5^. In this study, anchoring the SNP markers to the linkage groups of Asian seabass allows us to compare the genomic synteny between Asian seabass and other teleost species. A high level of conserved genomic synteny was observed between Asian seabass and European seabass (98.4%), followed by Nile tilapia (95.1%) and stickleback (90.8%), indicating close phylogenetic relationship with each other. Interestingly, in the comparison between Asian seabass and European seabass of the same number of chromosomes (24 pairs), the phenomenon of chromosome breakage and translocation was observed: LG14 of Asian seabass showed synteny to chromosomes 3 and 14 of European seabass. In the comparisons between Asian seabass and Nile tilapia (22 pairs of chromosomes) and between Asian seabass and stickleback (21 pairs of chromosomes), we identified evidence of chromosome fusions. For instance, LG9 and LG10 of Asian seabass were syntenic to chromosome 7 of Nile tilapia, while LG14 and LG20 of Asian seabass showed synteny to chromosome VII of stickleback. Likewise, we detected significant evidence of intra-chromosomal rearrangements for all the comparisons that interfered with the syntenic relationship at gene level due to gene loss and/or divergence in the process of evolution, especially in genome duplication^35^. However, only 5.6% of SNP markers in Asian seabass were identified in zebrafish and moreover no clear syntenic relationship was identified between the two species, suggesting complicated evolutionary events during the diversification of the two fish species^6^. The high level of conserved genomic synteny between Asian seabass and the species (Tilapia, stickleback) whose genomes were sequenced, makes the identification of candidate genes in mapped QTL easy in Asian seabass.

56.0% of the SNPs in the high-density genetic map were annotated to be associated with functional genes, suggesting that this map is a good start to identify genes within QTL^5^. Previous studies proved that extreme phenotypes supply a higher power in mapping QTL^36^,^37^. Therefore, in this study, we used 144 individuals with extreme growth from an F_2_ family for QTL mapping. We mapped a significant QTL on LG5. This result is consistent with our previous study where a major QTL was identified in LG5^28^. The significant QTL, which explained 16.0% of the total phenotypic variation for body weight, was refined into a 0.7 cM region in LG5 with an equivalent genomic length of 0.31 Mb. Five suggestive QTL were located in LG4, LG11, LG13, LG14 and LG15, respectively, with each explaining more than 10% of the phenotypic variation. Importantly, all the significant LOD regions of these five QTL were no more than 1.7 cM, corresponding to 0.75 Mb of genomic sequence^15^. However, in this study, neither significant nor suggestive QTL were detected in LG2, LG3, LG9, LG18 and LG24, where significant growth-related QTL had been mapped in previous studies^15^,^28^. Such inconsistent results are possibly due to the complex genetic architecture of growth traits, family-specific effects of QTL and different marker resolutions in LGs. In our previous study where the F_2_ family was used to detect growth-related QTL, 45 suggestive and 21 significant QTL were identified^28^, which were much more than that in this study, where a total of six QTL detected. We found that the phenotypic variance explained (PVE) by both suggestive and significant QTL in this study (10.5%–16.0%) were larger than that identified in our previous study (0.9%–12.0%). In particular, for some suggestive QTL in our previous study, the PVE were extremely low, less than 3%^28^. The major reason for such differences is possibly due to the difference in resolution of the genetic maps between the two studies, as the application of high-density mapping can reduce the false positives for QTL identified^38^. Altogether, these results suggest that the high-resolution genetic map supplies a good tool for accurate detection of QTL in Asian seabass.

The 0.7 cM region within the significant QTL in LG5 was further screened for candidate genes. By using genomic synteny analysis, six genes were identified within this narrow QTL region, among which *ACOX1* was the most promising candidate. *ACOX1* plays critical roles in lipid metabolism and has been shown to be associated with body weight gain and fat accumulation in mammals^39^,^40^. This gene was expressed in all the examined tissues and especially in the liver and kidney of Asian seabass. Interestingly, the expression in the fast-growing fish was significantly higher than that in the slow-growing fish in both liver and kidney. This is consistent with the function of this gene in catalysing fat deposition^40^, supporting the notion that *ACOX1* is a good candidate gene for growth. However, with the current data, we cannot rule out the involvement of other genes in the significant QTL for growth. Further detailed study on gene functions is required to understand the underlying mechanism of different growth among individuals.

In conclusion, a high-density genetic linkage map was constructed with 3321 SNPs using the ddRAD-seq approach. The marker density of this map was 0.52 cM. Comparative genomic analysis revealed a high level of syntenic relationship between Asian seabass and European seabass, Nile tilapia and stickleback. Using this map, a significant QTL for growth was detected in LG5 and five suggestive QTL for growth were located on five LGs. The *ACOX1* gene located in the significant QTL on LG5 was the most likely candidate responsible for growth. These data provide useful genomic resources for molecular dissection of complex traits, genome sequence assembly and genetic improvement in Asian seabass.

## Methods

### Ethics statement

All handling of fishes was conducted in accordance with the guidelines on the care and use of animals for scientific purposes set up by the Institutional Animal Care and Use Committee (IACUC) of the Temasek Life Sciences Laboratory, Singapore. The IACUC has specially approved this study within the project “Breeding of Asian seabass” (approval number is TLL (F)-12-004).

### Mapping population

The full-sib Asian seabass family used for linkage and QTL mapping was an new F_2_ population, which was generated with the same approach as the population used in our previous study^28^. Briefly, the F_1_ populations were obtained by mass crossing of 50 wild-captured fish from Southeast Asia. Dozens of pairs of F_1_ fish were then selected as parents according to their growth performance to generate F_2_ families by intercrossing. The offspring of F_2_ families were raised in a 40-ton indoor tank and fed twice daily under a standard feeding regime until nine months post hatch. The family showing a high level of phenotypic variations in growth-related traits was selected for linkage mapping. Growth traits including body weight (BW), total length (TL) and standard length (SL) were measured at the age of nine months^28^. Two parental samples and 142 progenies showing extreme phenotypic variations (BW ± s.d.): 528.5 ± 59.5 g vs 276.0 ± 66.9 g; TL: 32.9 ± 1.2 cm vs 26.5 ± 2.0 cm; SL: 28.5 ± 1.3 cm vs 22.3 ± 1.8 cm) were selected for construction of ddRAD libraries to increase the statistical power of QTL mapping. Genomic DNA was isolated from fin tissue using the salt precipitation method^41^ and was preserved at -80 °C until library construction.

### RAD library preparation and sequencing

Genomic DNA concentration was measured using Qubit® assays (Life Technologies, USA). RAD libraries were constructed using the double digest RADseq method with some modifications^9^. 200 ng of DNA from each sample was digested with 20 units of PstI-HF and MspI restriction enzymes (New England Biolabs, USA). Digested DNA fragments were examined by electrophoresis and were then ligated with barcoded adaptors^9^. Ligation products were pooled for size selection of 300–500 bp using Pippin Prep (Sage Science, USA) after clean up with QIAquick PCR Purification Kit (Qiagen, Germany). Selected libraries were then enriched by PCR with Phusion® High-Fidelity DNA Polymerase (New England Biolabs, USA). Finally, the libraries were cleaned up using QIAquick PCR Purification Kit (Qiagen, Germany) and quantified with KAPA Library Quantification Kits (Kapa Biosystems, USA) for paired-end sequencing on a NextSeq 500 platform (Illumina, USA), which produced single-end raw reads of 150 bp in length.

### SNP discovery and genotyping

The program *process_radtags* was employed to filter out raw reads with low quality scores and any uncalled base and also de-multiplex each sample data from the raw sequencing reads^34^. The reads were trimmed to 125 bp to rule out the sequencing errors at the end of each read. The program package Stacks (v1.21) was used for sequence mapping and stacks assembly, SNP discovery and genotyping^34^. A minimum of 20 times coverage for the parental samples were used to construct stacks and catalogue loci^34^. A maximum of two mismatches between stacks were allowed for catalogue construction. Catalogue loci with an extreme depth of short reads coverage were excluded^34^. For the progenies, stacks were assembled with a minimum coverage of five sequencing reads. The programs *sstacks* and *genotypes* were used to map each progeny data to the catalogue for SNP discovery and genotyping, respectively^34^. Loci with more than 20% missing data in the progenies were excluded from further analysis.

### Linkage map construction

Sex-specific and -averaged linkage maps were constructed using JoinMap4.1^42^. Prior to genetic mapping, the segregation patterns and ratio of SNP markers were examined. The SNPs were grouped into three categories according to type of segregation: in both parents (ab/ab), only in female (ab/aa or ab/bb) and only in male (aa/ab or bb/ab). SNPs showing significant segregation distortion with P < 0.01 in χ2 goodness-of-fit tests were excluded. Remaining markers were used for linkage group assignment with a threshold LOD score of 10.0. The number of calculated LGs and the number of markers assigned into each group showed no difference for LOD score ranging from 6.0 to 10.0. The positions of markers along LGs were determined using the regression mapping algorithm and Kosambi’s method with a recombination rate parameter of 0.40. The maps were drawn using MapChart v2.3^43^. As with our second-generation genetic map^15^, the order of LGs were determined by bridging the two genetic maps to the genomes of European seabass and Nile tilapia (*Oreochromis niloticus*) using comparative genomics (see below).

### Chromosomal recombination between sexes

The sex-specific maps were constructed using different markers and thus the recombination rates could not be directly compared using marker intervals^5^. The method developed by Gonen *et al.* (2014)^5^ was employed to investigate the difference in recombination rates between sexes. Briefly, each linkage group was divided into intervals of the same size and the number of intervals between each pair of male- and female-specific linkage group was equal. At least 10 intervals were defined for each linkage group. The percentage of markers mapping to each interval was calculated across each linkage group and compared between each pair of male- and female-specific LGs^5^.

### Annotation of RAD markers and comparative genomics

All mapped loci and paired-end contigs were aligned against the genome and annotation of European seabass (dicLab1), Nile tilapia (Orenil1.1) and stickleback (*Gasterosterus aculeatus*, Broad v1.0) using the program GMAP v13.8.19^44^ and SNPdat v1.0.5^45^ with default parameters. Considering the divergence between Asian seabass and each of the aforementioned three species, only loci with more than 70% of sequence length mapped to the reference genomes were retained. The other loci with no significant hits were further annotated using Blast2GO^46^ against all nucleotide databases. Only the most significant blast hit for each marker was used for annotation.

Genomic synteny was analysed between Asian seabass and each of the two perciforms, European seabass and Nile tilapia, and between Asian seabass and each of the two model teleost species, stickleback and zebrafish (*Danio rerio*, Zv9). The mapping and alignment were conducted as described above. Mapped RAD sequences with multiple targets were removed from further analysis in order to prevent misalignment. The number of significant alignments was calculated and genomic synteny was visualized using the program Circos v0.67^47^.

### Fine mapping of QTL for growth traits and identifying a candidate gene

The program MapQTL6^48^ was employed to detect QTL for phenotypic variations using both interval mapping (IM) and multiple QTL model mapping (MQM). The statistical significance of LOD threshold was determined by a permutation test of 1000 times for each trait. QTL with LOD scores exceeding the chromosome-wide LOD threshold at *P* < 0.05 were considered as suggestive, while those with LOD exceeding genome-wide LOD threshold at *P* < 0.05 and *P* < 0.01 were considered as significant and very significant, respectively.

The candidate genes within each QTL region were identified based on the annotation of SNP markers and genomic synteny between Asian seabass and European seabass. We identified a candidate gene, peroxisomal acyl-coenzyme a oxidase 1 (*ACOX1*), which is possibly responsible for growth traits (see below). Gene expression in 10 tissues, including brain, eye, gill, kidney, heart, liver, spleen, adipose, intestine and muscle, were investigated using real-time PCR according to our method^29^. PCR was conducted using KAPA™ SYBR® Fast qPCR Kits (Kapa Biosystems, USA) with iQ™5 Real Time PCR Detection Systems (Bio-Rad, USA). The primers for *ACOX1* were 5′-CCTGGTCAGAGCCTCAGATG-3′ and 5′-AGCAGTCCAGCCTGTAGGAA-3′. Elongation factor-1 alpha (EF1A) was used as internal reference^29^. The 2^−ΔΔCT^ method was employed to quantify the relative gene expression. The relative gene expressions were also detected between fast- and slow-growing Asian seabass (fast-growing fish, 169.7 ± 11.2 g vs slow-growing fish, 77.2 ± 7.4 g) using the above method. The fish used were from the same family with each group having at least three individuals.

## Additional Information

**How to cite this article**: Wang, L. *et al.* Construction of a high-density linkage map and fine mapping of QTL for growth in Asian seabass. *Sci. Rep.* **5**, 16358; doi: 10.1038/srep16358 (2015).

## Supplementary Material

Supplementary figures S1-S5

Supplementary Dataset S1-S4

## Figures and Tables

**Table 1:** Summary statistics of the sex-averaged genetic map of Asian seabass.

LG	Sex-averaged map		
	Mapped markers	Genetic length (cM)	Marker interval (cM)
1	52	43.47	0.84
2	186	73.60	0.40
3	166	65.39	0.39
4	147	52.29	0.36
5	188	53.22	0.28
6	158	75.23	0.48
7	118	83.90	0.71
8	151	74.54	0.49
9	158	55.67	0.35
10	113	122.49	1.08
11	107	71.29	0.67
12	184	77.47	0.42
13	201	68.24	0.34
14	100	80.26	0.80
15	177	41.52	0.23
16	178	64.05	0.36
17	116	48.39	0.42
18	161	55.79	0.35
19	131	81.08	0.62
20	70	34.71	0.50
21	98	66.41	0.68
22	76	63.29	0.83
23	155	60.98	0.39
24	130	64.39	0.50
Total	3321	1577.67	0.52

**Table 2:** Summary statistics of the significant and suggestive QTL for body weight and the nearest SNP to the peak of QTL in Asian seabass.

QTL	LG	Position	Highest	Explained	Nearest SNP
			LOD	variation	
Grow1	4	7.7–7.9	3.43	10.5%	Lca79807
Grow2	5	36.1–41.7	5.39	16.0%	Lca84235
Grow3	11	30.6–32.3	3.51	10.7%	Lca11069
Grow4	13	60.5–62.2	3.92	11.9%	Lca26478
Grow5	14	79.7–80.3	3.44	10.5%	Lca1879
Grow6	15	29.4–30.5	3.68	11.3%	Lca30784
