# Supplementary material for: Construction of a high-density linkage map and fine mapping of QTL for growth in Asian seabass
Source: Sci Rep. 2015 Nov 10;5:16358. doi: 10.1038/srep16358 (PMC4639833; doi:10.1038/srep16358)
Supplement: Supplementary figures S1-S5 [file srep16358-s1.pdf]

Construction of a high-density linkage map and fine mapping of QTL for growth in Asian seabass

Le Wang[1], Zi Yi Wan[1], Bin Bai[1], Shu Qing Huang[1], Elaine Chua[1], May Lee[1], Hong Yan Pang[1], Yan Fei Wen[1], Peng Liu[1], Feng Liu[1], Fei Sun[1], Grace Lin[1], Bao Qing Ye[1], Gen Hua Yue[1, 2, 3*]

[1]Temasek Life Sciences Laboratory, National University of Singapore, 1 Research Link, Singapore 117604

[2]Department of Biological Sciences, National University of Singapore, 14 Science Drive, Singapore 117543

[3]School of Biological Sciences, Nanyang Technological University, 6 Nanyang Drive, Singapore 637551

* Corresponding: genhua@tll.org.sg

Figure S1 Genetic lengths and marker distribution of 24 linkage groups in the female-specific map.

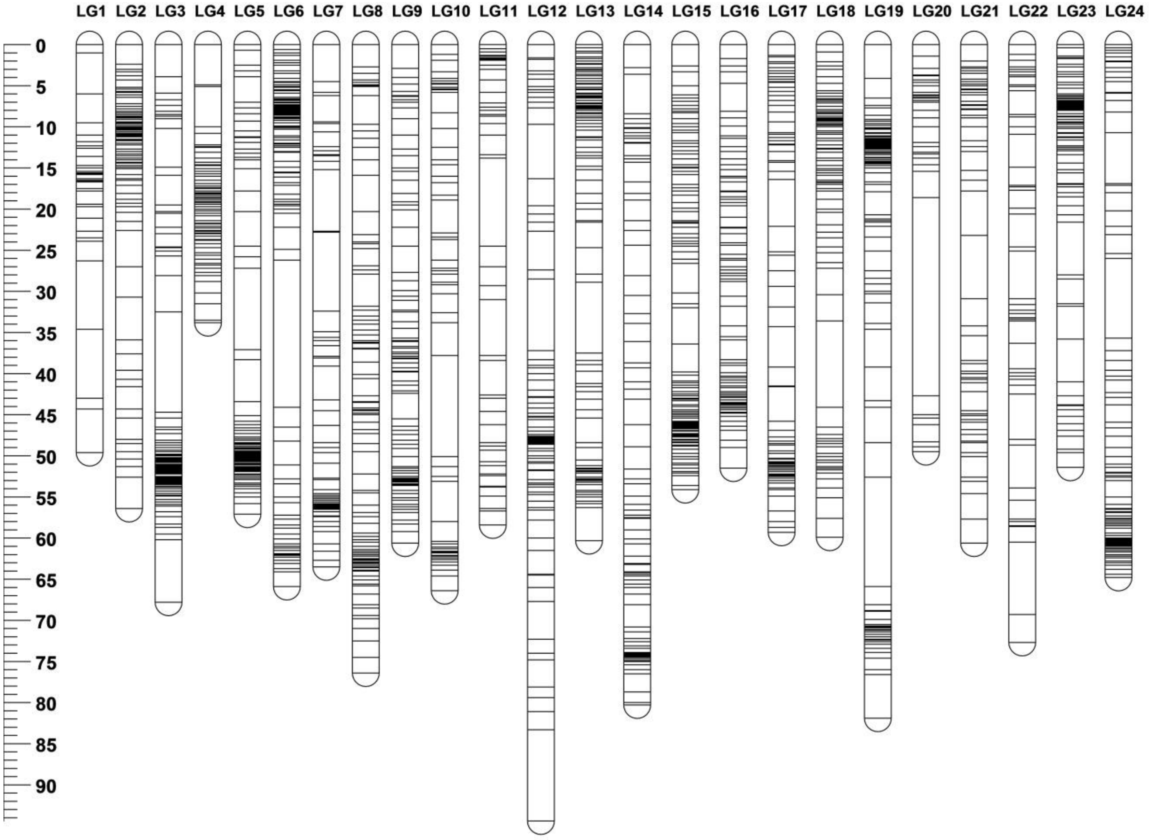

Figure S2 Genetic lengths and marker distribution of 24 linkage groups in the male-specific map.

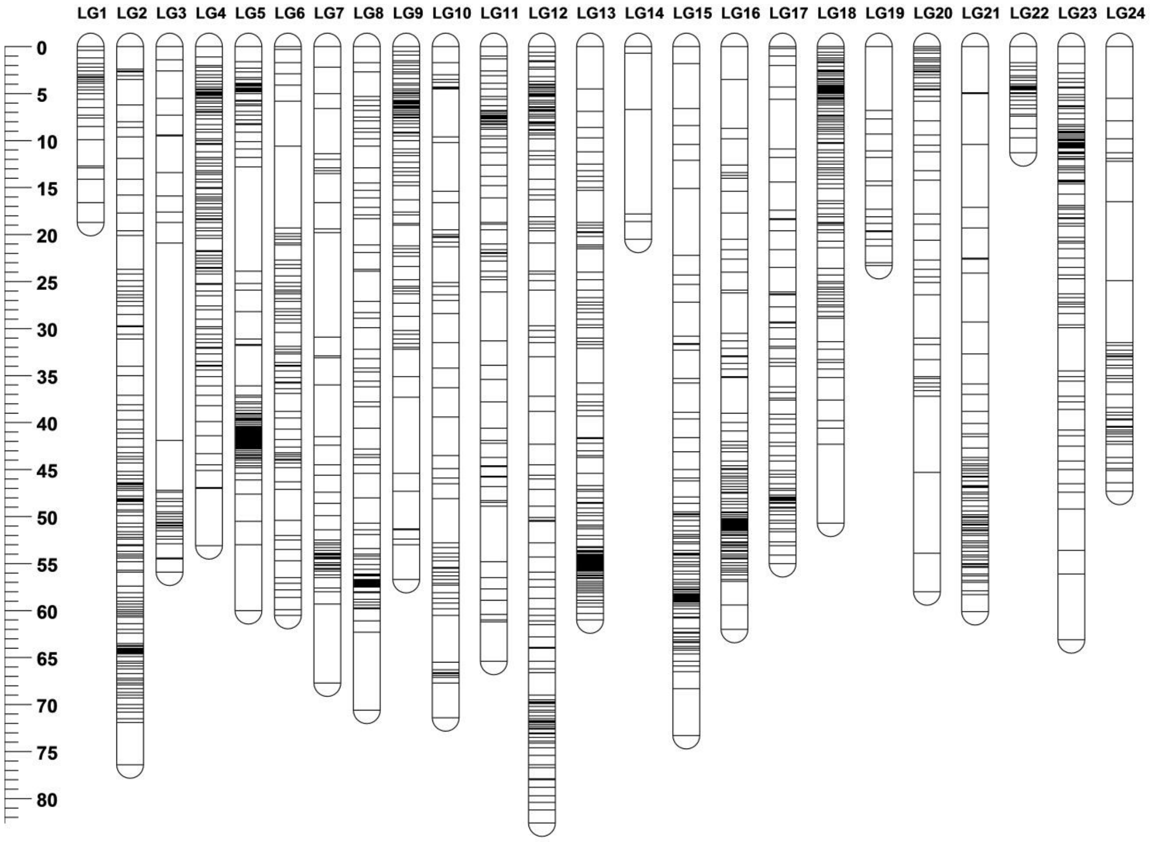

Figure S3 The distribution patterns of recombination events across each linkage group of sex-specific genetic maps of Asian seabass.

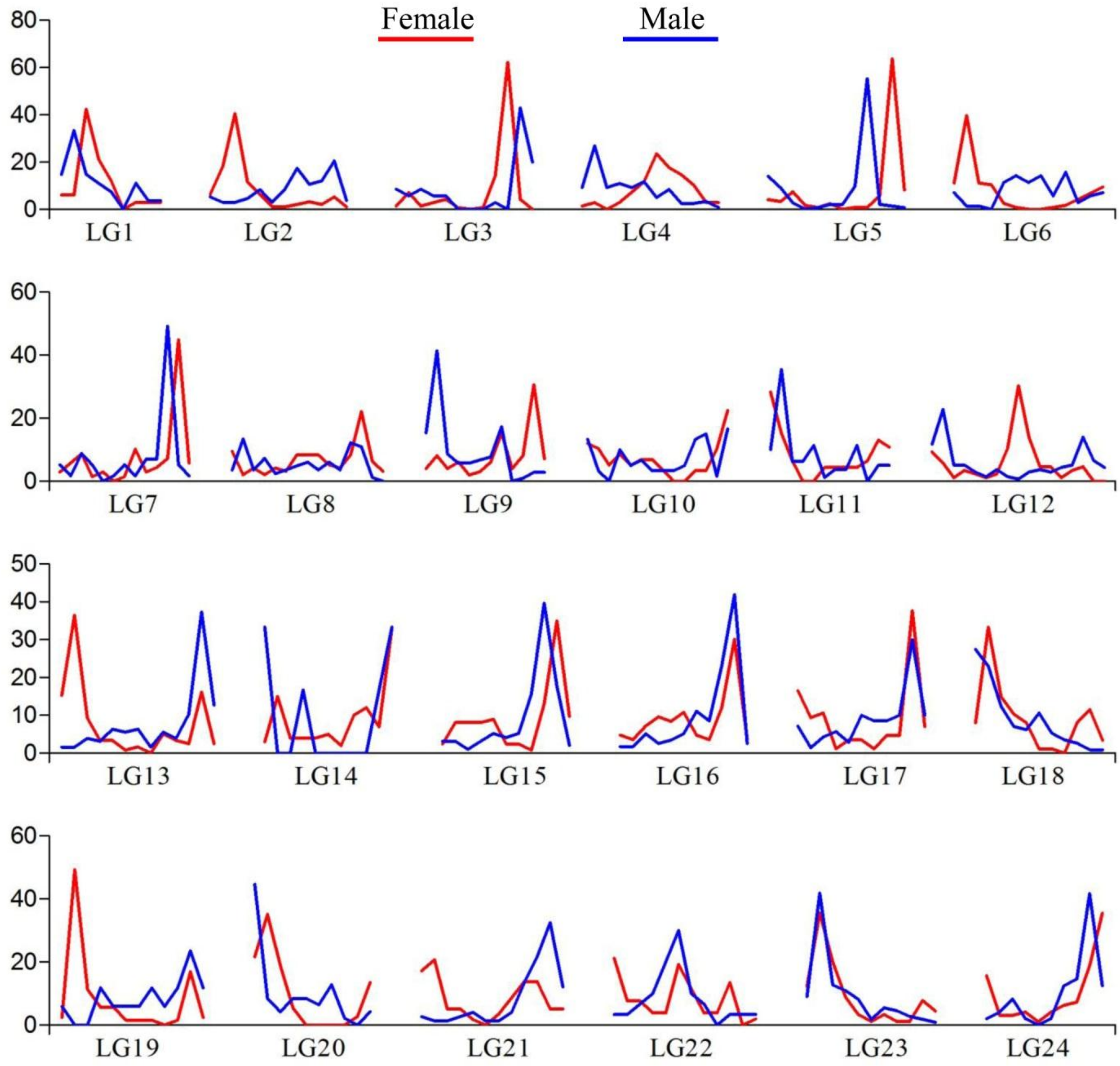

Figure S4 The number and percentage of annotated RAD markers in each linkage group of Asian seabass.

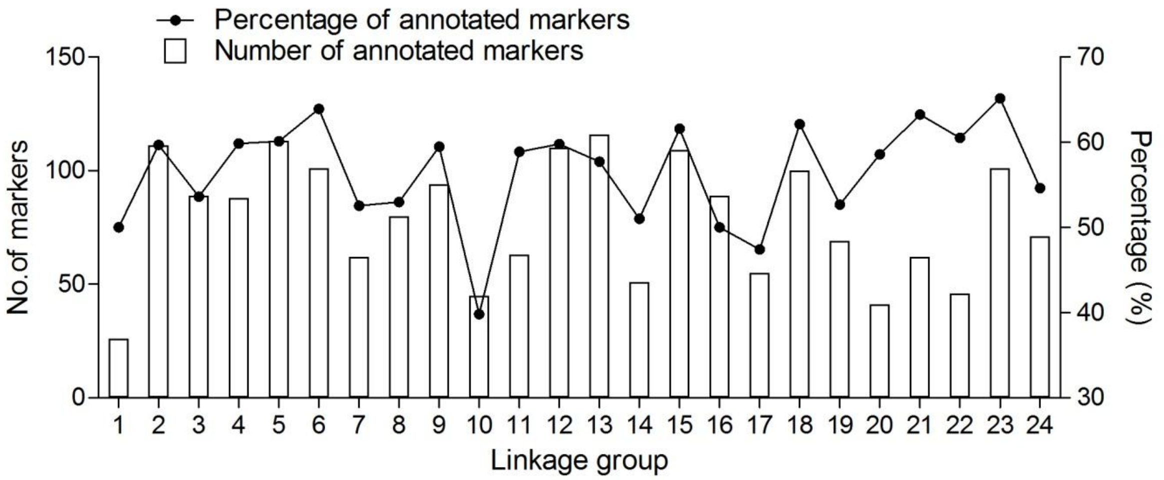

Figure S5 Genomic synteny visualized using Oxford grids between linkage groups of Asian seabass and chromosomes of European seabass, Nile tilapia and stickleback.

European seabass chromosomes (columns); Asian seabass LGs (rows)

	15	5	9	10	7	22-25	17	2	19	6	8	1A	20	3	16	24	4	X	12	14	13	11	1B	18-21
1	16																							
2	1	103			1					1														
3			87																	1				
4				83																				
5			2		84																			
6						78					1													
7							53															1		
8								65																
9									79															
10		1								41														
11											47													
12												83					1				1			2
13	1												92				1							
14														27						15				
15		1											1		74						1			
16								1								76								
17												1					44							
18		1																72						
19																			58					
20														1						29				
21																					47	1		
22																						36		
23																		1					78	
24	1																							50

Nile Tilapia chromosomes (columns); Asian seabass LGs (rows)

	16	1	22	18	6	20	15	2	7	4	5	12	3	11	23	17	19	10	14	13	8-24	9
1	17											1										
2		49		1					3		7						1					
3			52																			
4				46														1				
5					53																	
6						57																
7							37															
8					1			42			1											
9							2		57													
10					1	1			23											2		
11										32												
12					1						63											
13												67										
14	1	1											18									
15					1									77		1						
16	1	1												1	8							
17					1			1							15	5						
18																65						
19			1			2					2						40					
20			1										1			2		17				
21																		2	37			
22																1				18		
23																				1	60	
24													3									32

Stickleback chromosomes (columns); Asian seabass LGs (rows)

	XVI	II	X	III	IX	XII	XVIII	XIV	XIX	XI	XVII	XIII	VII	XX	I	VIII	IV	XV	VI	V	XXI
1	14		1												1		1				
2	1	57				1				1					1						
3			40		2	1				1				2							
4				39													1				
5					44					2			2		1						1
6		2				50										1					
7							39												1		
8	1							1									26		1		
9					1			50		1		2		1	1		1				
10						1			27						1						1
11										23			1								
12		1				3				1	38										
13							1	3				42	1						2		
14										2			24	2				1			
15		1	1				1		2				1	44	1		2		2		
16	4														36		1				
17															1	30					
18									1				1				34				
19																		34			
20													23				1		1		
21		1													32						
22																			22		
23	1										1		3							46	
24				1	1		1					1				1					25
